# The Different Welding Layers and Heat Source Energy on Residual Stresses in Swing Arc Narrow Gap MAG Welding

**DOI:** 10.3390/ma16114067

**Published:** 2023-05-30

**Authors:** Yuan Fang, Chunwei Ma, Guangkai Zhang, Yuli Qin, Wentao Cao

**Affiliations:** School of Materials Engineering, Shanghai University of Engineering Science, Shanghai 201620, China

**Keywords:** swing arc, narrow gap MAG welding, residual stress, ABABQUS, blind hole method

## Abstract

In this paper, in order to reduce the time cost of prediction experiments in industry, a new narrow gap oscillation calculation method is developed in ABAQUS thermomechanical coupling analysis to study the distribution trend of residual weld stresses in comparison with conventional multi-layer welding processes. The blind hole detection technique and thermocouple measurement method verify the reliability of the prediction experiment. The results show that the experimental and simulation results have a high degree of agreement. In the prediction experiments, the calculation time of the high-energy single-layer welding experiments is 1/4 of the traditional multi-layer welding. Two welding processes of longitudinal residual stress and transverse residual stress distribution trends are the same. The high-energy single-layer welding experiment stress distribution range and transverse residual stress peak are smaller, but the longitudinal residual stress peak is slightly higher, which can be effectively reduced by increasing the preheating temperature of the welded parts. This implies that in the specific case of increasing the initial temperature of the workpiece, the use of high-energy single-layer welding instead of multi-layer welding to study the residual stress distribution trend not only optimizes the weld quality but also reduces the time cost to a large extent.

## 1. Introduction

Thick steel plate pressure-bearing equipment is a pressure boundary subject to specific temperatures, pressures, and corrosive media and is widely used in mechanical engineering, nuclear power, pharmaceutical, and aviation. Welding is an essential method of manufacturing pressure-bearing equipment [1,2,3]. However, due to the uneven temperature distribution and large thermal gradients between the different areas of the workpiece in the process of heating and cooling of the experiment, resulting in local expansion and contraction of the metal components, the elastic yield limit can only constrain minimal mismatch strain. These large enough mismatch strains lead to plastic deformation of the part, which greatly affects residual stress during processing [4,5]. In addition, during high-temperature melting and cooling solidification, the intermediate zone is constrained by the surrounding environment, generating tremendous tensile residual stresses altering the mechanical properties of the workpiece connection, and the structure is deformed, cracked, creep damage and other adverse effects, affecting the working life of the workpiece [6,7,8,9,10]. Therefore, the inevitable welding residual stress plays an essential role in the connection and pressure bearing of the equipment; effective control and study of the residual stress distribution can ensure the integrity of the pressure equipment

The traditional welding method mainly adopts a large area of V-shaped slope when welding large thick plates, which has high operation requirements, a small bearing area, and a non-smooth workpiece matrix material and joint transition. The double V-bevel was improved on the V-bevel, and Mehmet et al. [11] optimized the joint by varying the three welding parameters of the break angle, current, and voltage to determine the optimal parameters of the average tensile strength and elongation for a better study of the mechanical properties of the carbon steel workpiece joint. The narrow gap GMAW in thick plate welding mainly uses a smaller tangent plane area of the I-shaped slope, which has high productivity, low consumption of welding material, residual stress and deformation, and other advantages [12], and is a promising thick plate welding method. However, the side wall of the workpiece with a narrow gap GMAW tends to be incompletely fused during processing, forming welding blemish. The emphasis in solving this problem is to ensure that the arc stays on the sidewall for enough time to provide sufficient sidewall heating. Therefore, scholars from various countries have developed various forms of narrow gap GMAW. The more common narrow gap GMAW are rotating narrow gaps [13,14], double wire narrow gaps [15,16], and oscillating narrow gaps [17,18,19]. This experiment uses swing narrow gap GMAW because the swing speed, swing amplitude, and sidewall dwell time of the swing arc are controllable, its control parameters are more and better adaptable; compared with rotary narrow arc gap GMAW, swing arc welding in the wire and sidewall angle is generally more extensive, the arc has a short dwell in the sidewall, so the sidewall is more fully heated; compared with double-wire welding, the energy of swing welding heat source releases is lower [20], the melt pool volume is smaller, the melt pool is less likely to flow and is suitable for all-position welding. Therefore, this experiment uses swing narrow gap GMAW, a highly efficient welding technique with some application prospects.

The exploration of residual stress field measurement technology began in the 1930s and has since developed into dozens of measurement methods, roughly divided into two measurement methods: mechanical measurement and physical measurement [21]. The blind hole method is portable and inexpensive compared to other residual stress measurement methods. It is the most frequently used and recognized method for measuring residual stress [22]. However, the detection of residual stress using the blind hole method, the detection process by the plastic deformation of the material, heat, operator proficiency, and other factors, so that its measurement accuracy is more constrained, so with the maturity and development of computer and numerical simulation calculation methods, the use of finite element simulation method is more intuitive and efficient to study the welding process.

Behrouz et al. [23,24,25] repeatedly used simulations and experiments to certify the stir friction welding method. A very in-depth study of the thermal cycle, mechanical properties, and microstructure of vibration and non-vibration stir friction welding was systematically analyzed and studied by optimizing the welding speed, rotational speed, tilt angle, shoulder diameter, and the number of welding paths, respectively, and according to the data obtained it was found that the selection of optimized parameters could better reduce the highest value of residual stress, the elements at the joint were more uniformly distributed, the thermal properties were better, and the workpiece weld quality was significantly improved. Dean Deng et al. [26] found that the thermoelastic method can effectively simulate heat transfer, metal melting and solidification, residual stresses, and weld deformation due to phase changes during welding. Hashemi et al. [27] exploited a new welding applied load model for two-dimensional and three-dimensional simulation to detect the step-by-step law of the temperature field and stress field. The three-dimensional simulation test results are more accurate, and the two-dimensional simulation test efficiency is higher. Liu et al. [28] verified a new mechanical model with experimental results, and the related changes of the welding experiments on the deformation and residual stress of aluminum plates were analyzed. Kartal et al. [29] explored the variation trend of the stress field of thick plate with different welding processes of welding experiments with varying numbers of welds depending on the inverse intrinsic technique and contour method. The conclusions demonstrate that the highest tensile stress appears in the workpiece connection with a low-energy heat source, and the ultimate tensile stress field appears in the areas on both sides of the workpiece connection with a high-energy heat source. Xu Shugen et al. [30] used sequential coupled finite elements to predict the conversion of the temperature field in the welding experiment to calculate the converting trend of the stress field in the workpiece connection and both sides of the area but also studied that the higher the heat energy of the workpiece before the experiment, the more stable the stress field distribution trend is. Welding heat sources with different energies have little effect on the distribution trend of the stress field. The linear heat source is used instead of the oscillating heat source to improve the simulation calculation efficiency by Zhang et al. [31]. However, it does not consider that the linear heat source has a small heating range and a weld with a width of 20 mm is unsuitable for single-pass welding.

In the present work, two single- and five-layer simulation models with body coordinates of 20 mm thickness were established using ABAQUS 2017 software using thermodynamic coupling calculations only by increasing the current and voltage to obtain a low-energy and high-energy heat source with a heat input of 1820 J/mm and 3366 J/mm and act on the single-layer single-pass and five-layer single-pass narrow gap swing welding models, respectively, to study the distribution of the residual stress field. In order to verify the accuracy of the predicted results, the thick plate using five single-layer swing welding experiments, installed at 100 mm from the edge of the workpiece thermocouple and blind hole detection technique to explore the distribution of temperature and stress fields. Finally, by comparing finite element simulation and inspection experiments, the stress field distribution is explained in principle, and a new high-energy heat source prediction method is developed instead of the calculation of multi-layer welding in the simulation to reduce the time cost of prediction experiments performed in the industry.

## 2. Experimental Procedure

**Figure 1:**
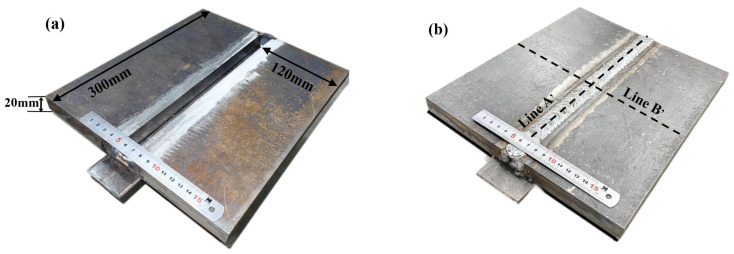
The images of the MAG welding plate. (**a**) Plate is before welding. (**b**) Plate is after welding.

Q235A low carbon steel rolled steel pipe and steel plate’s toughness and plasticity are reasonable, and there is an excellent elongation, which has good welding properties and hot workability to manufacture various welded structures. Copper-plated wire of low-alloy steel effectively prevents the tendency of porosity and slagging of the wire because of the copper-plated surface treatment, good electrical conductivity during welding, smooth wire feeding, less spatter, and beautiful weld seam. Furthermore, the addition of a specific ratio of helium in the shielding gas can improve the welding quality and optimize the shape of the weld seam [32,33]. As shown in Figure 1, the welded base material is two pieces of Q235A steel plate with a *Z*-axis direction size of 20 mm, the welded specimen size is 300 mm × 120 mm × 20 mm, the weld width is 20 mm, five-layer single pass welding without beveling, the welding wire metal is H08Mn2SiA solid wire with a sectional dimension of 1.2 mm, the area around the weld is scrubbed with the organic solution, when welding it is brushed with a steel wire ball to remove oxides. The components are welded with fixed blocks at the arc starting and closing positions, and arc plates are welded at the bottom of the weld. The performance attribute and chemical composition of the thick plate material and the filling material are displayed in Table 1 and Table 2.

**Figure 2:**
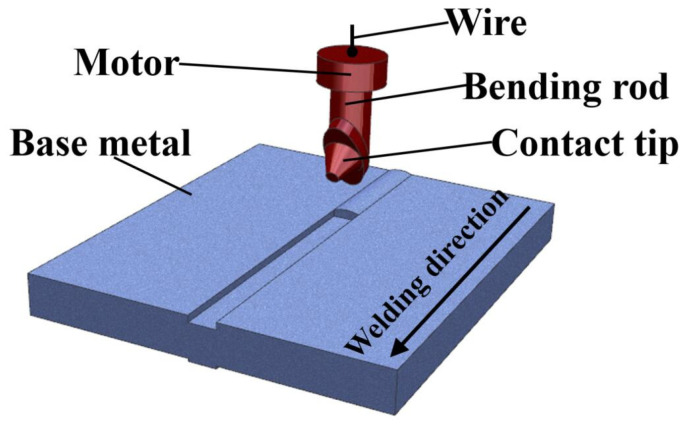
Low-energy swing heat source experimental diagram.

Figure 2 shows an experimental scene diagram of swing arc welding with five weld passes in which the metal welding wire is fed through the mechanical arm contact. Through the experimentation, the mechanical arm contact is rotated at an angle around the mechanical arm center of the rod, thus controlling the constant angle of the arc during the swing, with the electrode swinging at 450 rad/min and a swing angle of 145°, the conductor wire is at an angle of 10° to the welding torch. Yu Dongliang et al. [34] conducted a very systematic study of the weld parameters using neural networks through an in-depth learning approach and many experiments to investigate the geometric parameter equations of the GMAW joint. Therefore, according to the geometric parameter equation, the welding gun advance speed was selected as 18 cm/min, the experimental current is 280 A, the practical voltage is 28 V, the welding flow rate is 10 L/min, and the welding metal welding wire forward speed is 11.8 m/min.

**Figure 3:**
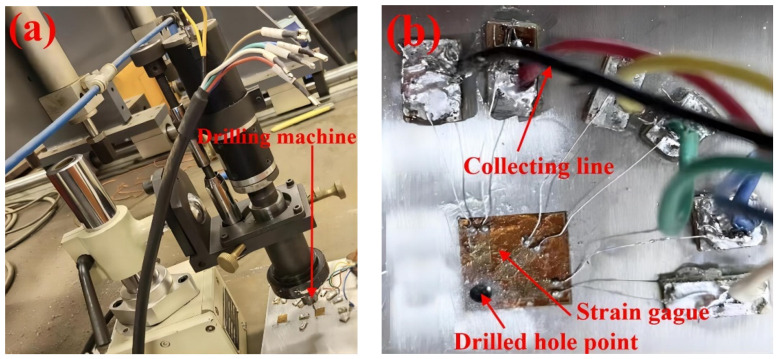
(**a**) Drilling process, (**b**) The connecting of the strain gage.

The experimental procedure is displayed in Figure 3. First, grind the weldment clean. The strain gauges are attached to the parts to be measured, and the leads of the strain gauges and the shielding wires are soldered to the corresponding terminals with a soldering iron; then, the shielding wires are connected to the strain gauges, the strain gauges are switched on and started, the relevant parameters are assigned in terms of the material to be tested and the type of strain gauges, warm-up, zeroing, punching, recording the corresponding displacement and strain due to the stress release in part around the holes, measuring the distance before and after the drilling of the calibration points around the holes using a mechanical extensometer, and solving through Equations (1)–(3) [35].

(1) $$\sigma_{1}=\frac{1}{4A}\left(\varepsilon_{1}+\varepsilon_{3}\right)-\frac{1}{4B}\sqrt{{\left(\varepsilon_{1}-\varepsilon_{2}\right)}^{2}+{\left(2\varepsilon_{2}-\varepsilon_{1}-\varepsilon_{3}\right)}^{2}}$$

(2) $$\sigma_{2}=\frac{1}{4A}\left(\varepsilon_{1}+\varepsilon_{3}\right)+\frac{1}{4B}\sqrt{{\left(\varepsilon_{1}-\varepsilon_{2}\right)}^{2}+{\left(2\varepsilon_{2}-\varepsilon_{1}-\varepsilon_{3}\right)}^{2}}$$

(3) $$\tan2\theta =\frac{2\varepsilon_{2}-\varepsilon_{1}-\varepsilon_{3}}{\varepsilon_{1}+\varepsilon_{3}}$$

where σ_1_ and σ_2_ represent the maximum and minimum principal stress of residual stress before drilling. ε_1_, ε_2_, and ε_3_ illustrate the release strains measured at 0°, 45°, and 90°, respectively. A and B represent the strain discharge coefficient obtained by the calibration test, 10^−7^ mm^2^/N. θ is the angle between σ and ε.

**Figure 4:**
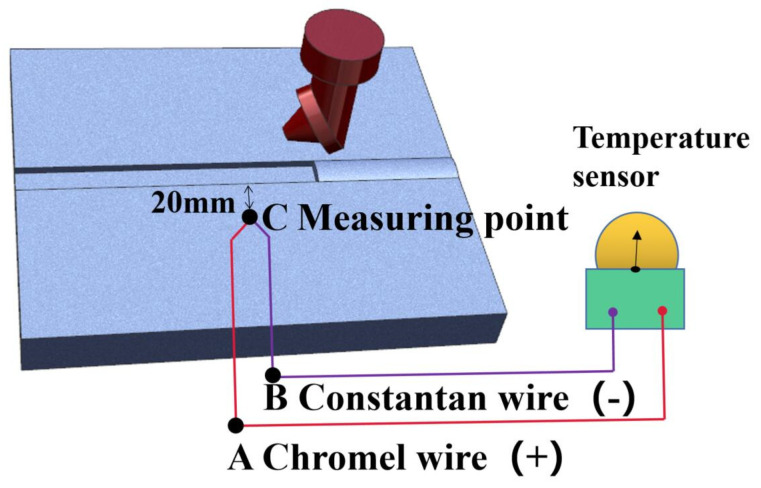
The working diagram of thermocouple.

A built-in DC thermocouple welding machine is used to test the thermal welding cycle of the weldment. The experiment is shown in Figure 4. Two colors represent different metal materials. The A and B ends are used for temperature measurement in a typical environment. C is heated for the test point. Due to the thermoelectric effect, the temperature between the three ends of A, B, and C is different, resulting in a potential difference. The material of the thermocouple is chromate. Finally, the temperature in the workpiece is measured by the instrument.

## 3. ABAQUS Simulation

**Figure 5:**
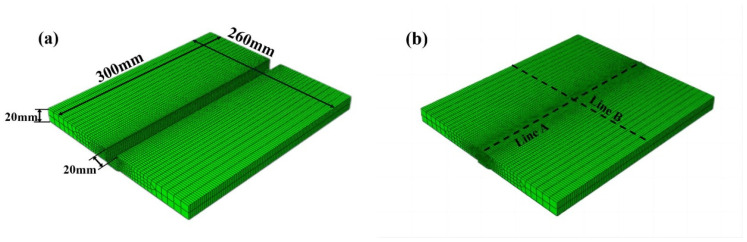
Simulated workpiece model. (**a**) The initial state of the model before welding. (**b**) The final state of the model after welding.

Simulation software ABAQUS 6.6-3, ANSYS/EMAG, SYSWELD, and MARC are widely used to predict the thermal effects, mechanical behavior, and plastic deformation in the welding process, where the thermomechanical coupling calculation method of ABAQUS can more accurately simulate the mechanical properties of the workpiece and more easily edit the material properties and optimize the operation process through the script language [36,37,38,39]. The prediction experiment establishes two ABAQUS simulation models with the actual weld’s exact dimensions. The experiment uses the thermodynamic coupling calculation method to simulate the residual stress distribution pattern of single-layer and five-layer welds. The life and death cell technique is used to recover more realistic scenarios of solder-filled welds. The “model change” function is used to extinguish and activate the welding channel of each layer, which can produce more efficient and accurate results. The mesh is edited to be non-uniform, with a fine mesh near the joints with significant temperature variations and a relatively coarse mesh at the edges of the workpiece where the temperature gradient is gentle to ensure the calculation efficiency can be improved while controlling the accuracy [40]. The grid model is identical to the eight-node hexahedral temperature-displacement coupled cell C3D8RT. Its thermal-force behavior is simulated by “complete coupling,” the coupling term is considered in the fundamental equations, and the stress field affects the temperature field. The physical and thermal properties of the experimental material are exhibited in Table 3 and Table 4, and the simulation prediction model is shown in Figure 5.

Goldak [41] used a dual elliptical heat source to simulate the three-dimensional heat input during the experiment, and the user subroutine was invoked through ABAQUS DFLUX to move the load in the specified direction. In narrow gap oscillation MAG welding, the forward direction of the torch, the angle of the wire swing, and the distance between the torch and the workpiece surface all have prominent results on the distribution of the temperature field. To control the influence of these constraints in the experimental process and simulate the testing process more realistically, the double elliptical heat source model is selected in this experiment because the direction of the heat output is constantly changing with the deflection of the arc.

The heat source parameters are expressed in Equations (4) and (5) [31]:

(4) $$q_{f}=\frac{12\sqrt{3}\eta I}{\pi \sqrt{\pi }ab(c_{f}+c_{r})}exp\left[-\frac{3X_{n}^{2}}{a^{2}}-\frac{3Yn^{2}}{b^{2}}-\frac{3Z_{n}^{2}}{c_{f}^{2}}\right]$$

(5) $$q_{r}=\frac{12\sqrt{3}\eta IU}{\pi \sqrt{\pi }ab(c_{f}+c_{r})}exp\left[-\frac{3X_{n}^{2}}{a^{2}}-\frac{3Yn^{2}}{b^{2}}-\frac{3Z_{n}^{2}}{c_{r}^{2}}\right]$$

Here $q_{f}$ is the heat flow function for the front ellipsoid, $q_{r}$ is the back ellipsoid; η, I and U are the specific heat ratio, and welding condition, respectively, and $c_{f}$, $c_{r}$, a and b are all model ellipsoidal heat source size parameters.

**Figure 6:**
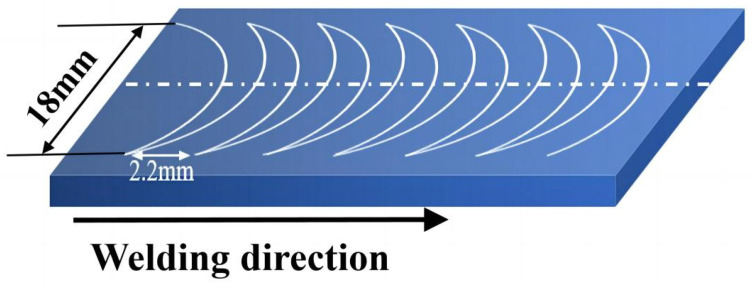
Heating center swing moving diagram.

Figure 6 is a schematic diagram of the load-moving path, a compound motion of linear motion, and circular arc oscillation. The welding gun makes a uniform motion of 3 mm/s along the centerline of the connection, and the metal wire makes a constant circular motion of 450 rad/s with the centerline as the center. When the lateral coordinates of the heat source during the movement coincide with the lateral coordinates of the starting arc position, it marks the completion of a motion cycle, and the whole experimental process consists of multiple cycles. The welding speed, swing angle, swing frequency, and swing radius determine the heat source trajectory. The heat input is determined by current, voltage, welding speed, and coefficient *η*. Increasing the heat input without changing the heat source trajectory can only be obtained by increasing the current and voltage for low-energy and high-energy heat sources with a heat input of 1820 J/mm and 3366 J/mm, and acting in single-layer single-pass and five-layer single-pass narrow-gap oscillation welding models, respectively.

**Figure 7:**
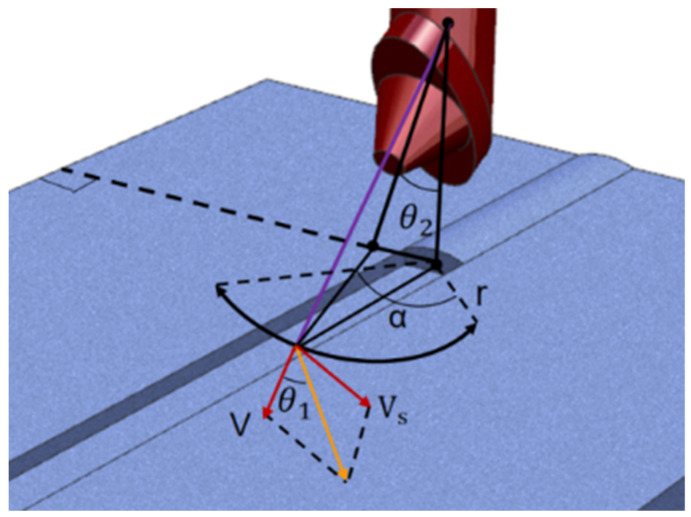
Detailed parameter diagram of swing motion.

Figure 7 displays the correlation between the torch axis position and the arc swing radius and angle in the experiment. This is because the heating scope of the workpiece is proportional to the swing angle of the welding torch. When the sweep angle of the welding torch increases, the heating area of the workpiece is larger, the heating time of the weld edge is longer, the deposited material is more, and the shape of the welding channel formed by the molten pool is better. Lower swing frequencies result in longer dwell times near the sidewalls and overheating of the molten base metal. Because there is a certain length between the mechanical arm contact and the workpiece bottom surface, the heating center has a long contact time with the upper side wall, and the heat of the lower part of the side wall cannot lead to the melting of the molten pool. The greater the cover tension on the surface of the workpiece, the droplets fall to the bottom to cool and solidify into a weld. As the arc swing frequency increases, the formed molten pool has more cooling time. Therefore, the weld shape formed by molten metal liquid is flat. The trajectory of the arc is described using a periodic function of time, and the following parametric equation can represent its moving path (6)–(9).

(6) $$t_{1}{\leq t\leq t}_{2}\;\left\{\begin{array}{c} x=r\cos\left(\alpha +15\right)\\ y=Vt+r_{0}\sin\left(V_{s}t+15\right) \end{array}\right.$$

(7) $$t_{2}{\leq t\leq t}_{3}\;\left\{\begin{array}{c} x=-r\sin\left(V_{s}t\right)\\ y=Vt+r\cos\left(V_{s}t\right) \end{array}\right.$$

(8) $$t_{3}{\leq t\leq t}_{4}\;\left\{\begin{array}{c} x=-\mathrm{rcos}\left(\alpha +15\right)\\ y=Vt+r\sin\left(V_{s}t+15\right) \end{array}\right.$$

(9) $$t_{4}{\leq t\leq t}_{5}\;\left\{\begin{array}{c} x=r\sin\left(V_{s}t\right)\\ y=Vt+r\cos\left(V_{s}t\right) \end{array}\right.$$

(*x*, *y*, *z*) are the coordinates of the arc heating center point, the *Z* coordinate remains unchanged during each layer of welding, *t*_1_ to *t*_5_ is a section of several periods; *r*, *V*, *V_s_*, *α* are the motion parameters of the welding torch during the experiment.

## 4. Results and Discussion

### 4.1. Low-Energy Heat Source Thermal Analysis

**Figure 8:**
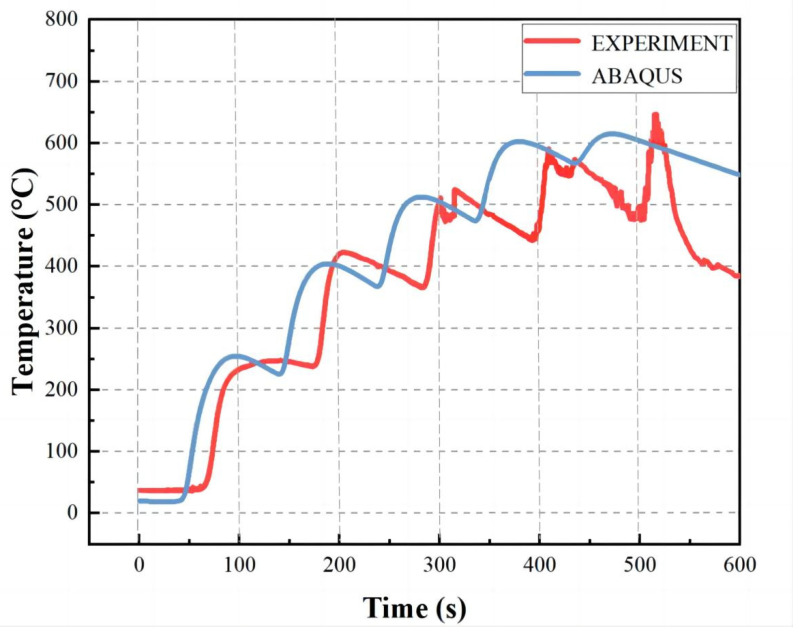
Thermal analysis experimental diagram.

To study whether the simulation results are accurate, Figure 8 shows the experimental thermal analysis curve of the test point C at 100 mm from the edge of the workpiece; the simulation and experimental results are consistent. The two curves show five superimposed peak trends. The maximum temperature value of the next layer of the motion path is higher than the maximum temperature value of the previous layer, and the five peak slopes formed are steep. The peak value of the experimental curve appears more because this article uses a five-layer single-pass welding. Each layer of the weld is shorter, the previous layer has not been cooled, and the next welding layer is started; the latter weld is welded under the preheating condition of the prior layer. During the experiment, the workpiece is welded by swing welding, and the periodically repeated movement of the arc at different positions means that some areas are repeatedly heated and cooled in a short period so that the experimental curve will have more peaks.

### 4.2. Residual Stress on Simulation and Experiment

**Figure 9:**
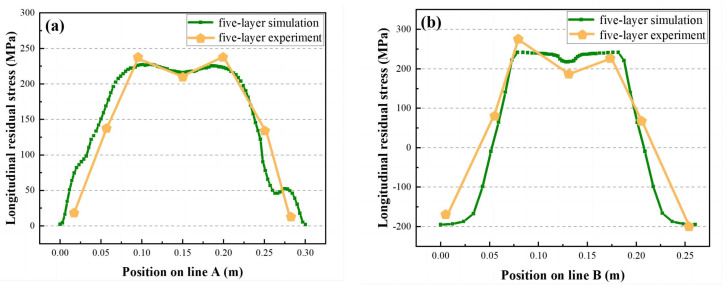
Prediction and experimental results. (**a**) Longitudinal residual stress distribution in line A. (**b**) Distribution of longitudinal residual stresses in line B.

In the five-layer one-way welding experiment, the residual stress distribution of the workpiece is uneven, and it is hard to detect the stress field distribution on the surface of the thick plate. Therefore, in this part of the experiment, the ABABQUS prediction results and the blind hole measurement technology are applied to compare the longitudinal stress field (σ_x_) at the two detection lines. In Figure 9a, the σ_x_ distributions of the A-line and A′-line obtained from simulation and experiment are consistent and belong to the tensile state. The difference between the highest tensile stress measured in the experiment of 240 MPa and the predicted experiment is less than 5%. In Figure 9b, the σ_x_ distributions of the B-line and B′-line obtained by simulation and experiment are symmetrically distributed along the centerline of the workpiece joint. The distribution pattern is compression, tension, and recompression, and the difference between the highest tensile stress measured in the experiment of 265 MPa and the predicted experiment is less than 6%. The experimental results are almost consistent with the predicted results, which indicates the high reliability of the two predicted experiments.

### 4.3. Stress Field Distribution

The variation of the residual stress field of the medium and thick plate workpieces is determined by the melting welding method, but most are two-way stress, that is, plane stress state. Therefore, the stress field in the thickness direction is microscopic. Only in the weld of the rear section of the large structure the stress field in the thickness direction has a higher value, so the stress in the thickness direction is not considered in this simulation. In addition, because the peak value of the longitudinal residual stress is on the middle line of the weld, the transverse peak value is smaller than the longitudinal peak value. This experiment studies the plane stress field distribution of line A and line B, and the two lines are shown in Figure 5.

#### 4.3.1. Distribution of Longitudinal Stress Field

**Figure 10:**
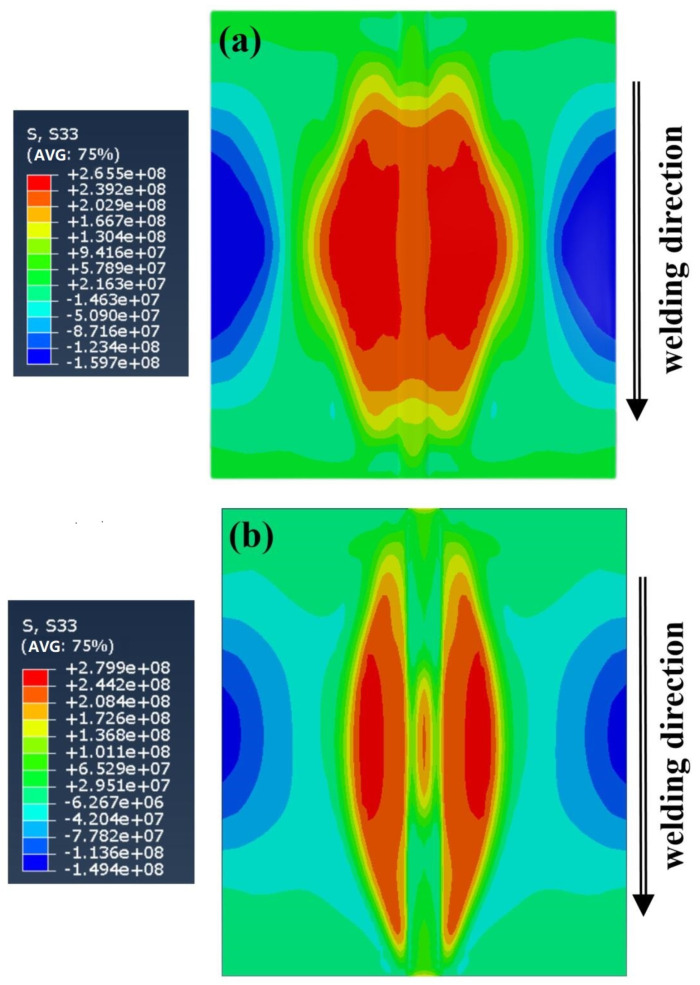
Longitudinal stress field distribution: (**a**) is low-energy heat source, (**b**) is high-energy heat source.

Figure 10a is a longitudinal stress field (σ_x_) distribution cloud map of the five-layer welding experiment, and Figure 10b shows a single-layer welding experiment. The longitudinal stress field distribution trends of the two welding processes are the same. The workpiece joint and the heat-affected zone of the workpiece show a high tensile stress trend, reaching the yield limit of the base material, the arc starting position, the arc extinguishing position, and the edge of the workpiece show a compressive trend, the highest compressive stress field is located on both sides of the workpiece, balanced with the tensile stress in the middle region. This distribution is because the metallic material receives uneven local heating. When the weld joint melts, the surrounding material inhibits the expansion of the highly heated part and produces compressive deformation. During the cooling process, the amount undergoing plastic deformation is restricted by the surrounding material and is not free to contract, resulting in a tensile force field.

The tensile range and compression range of the single-layer welding experiment were narrower than the five-layer welding experiment, indicating that the predicted experiment of single-layer welding is of higher processing quality. However, the tensile peak of single-layer welding is more heightened than five-layer welding. The reason for this phenomenon is that in single-layer welding in the experimental process using a high-energy heat source, the same speed of movement, swing arc, swing frequency, and single-layer welding temperature gradient increases, and the contact time with the side wall of the workpiece is extended, the workpiece melts more widely, the resulting peak stress is prominent.

**Figure 11:**
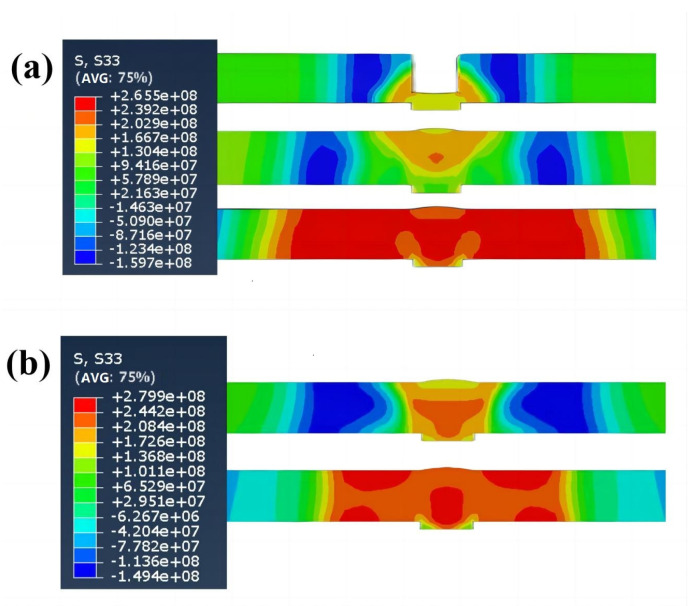
The σ_x_ diagram of the middle along the workpiece B line cutting: (**a**) is the five-layer experiment, (**b**) is the single-layer experiment.

Figure 11a shows the straining process of the thermal elastic-plastic σ_x_ of the metal material around the five-layer welding moving swing heat source. At the end of the first layer of the experiment, the welded joint shows a tensile trend, the highest stress value appears on the measuring wall, and both sides of the molten pool are compressed. At the end of the fifth layer of welding, the tensile state tends to expand to both sides, and the compression state on both sides of the molten pool is transformed into the tensile state, crossing the elastic unloading zone; after the experiment is completed, it is cooled to the final state at ambient temperature, and the tensile plastic zone in the center of the welded joint and the accumulated compressive plastic strain on both sides evolve into an elastic unloading zone.

Figure 11b shows the straining process of the thermal elastic-plastic σ_x_ of the metal material around the single-layer welding moving swing heat source. At the end of the first layer of the experiment, the welded joint shows a tensile trend, the highest stress value appears on the workpiece connection, and both sides of the molten pool are compressed. After the experiment is completed, it is cooled to the final state at ambient temperature; because there is no multi-layer superimposed thermal effect, the range of the elastic unloading zone is smaller than that of the five-layer experiment.

**Figure 12:**
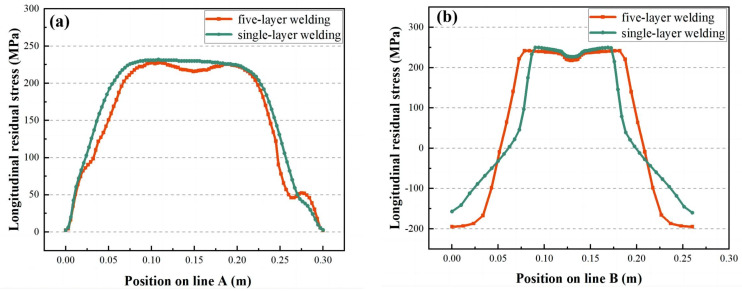
The stress field contrast diagram of high-energy heat source and low-energy heat source: (**a**) is the longitudinal stress field of A line, (**b**) is the longitudinal stress field of B line.

Figure 12 displays the distribution trend of the σ_x_ of line A and line B under different energy heat source loading. The changing trend of the stress field of the high-energy heat source and the low-energy heat source is consistent and symmetrical about the center of the welded joint. The stress field of line A is a tensile trend from arc starting to arc extinguishing. The maximum tensile state of approximately 230 MPa is located in the middle area of 80 mm from the two ends, and the closer the tensile trend is to the margin, the smaller the tensile stress is. The area near the arc starting and extinguishing of the stress field of line B is a compression trend, and the middle part is a tensile trend. The maximum tensile stress for a single-layer weld is approximately 255 MPa. The central area is 75 mm from the edge of the workpiece. The maximum tensile stress for a five-layer weld is approximately 250 MPa, a central area 85 mm from the edge of the workpiece, 13% wider than for a single-layer weld.

#### 4.3.2. Distribution of Transverse Stress Field

**Figure 13:**
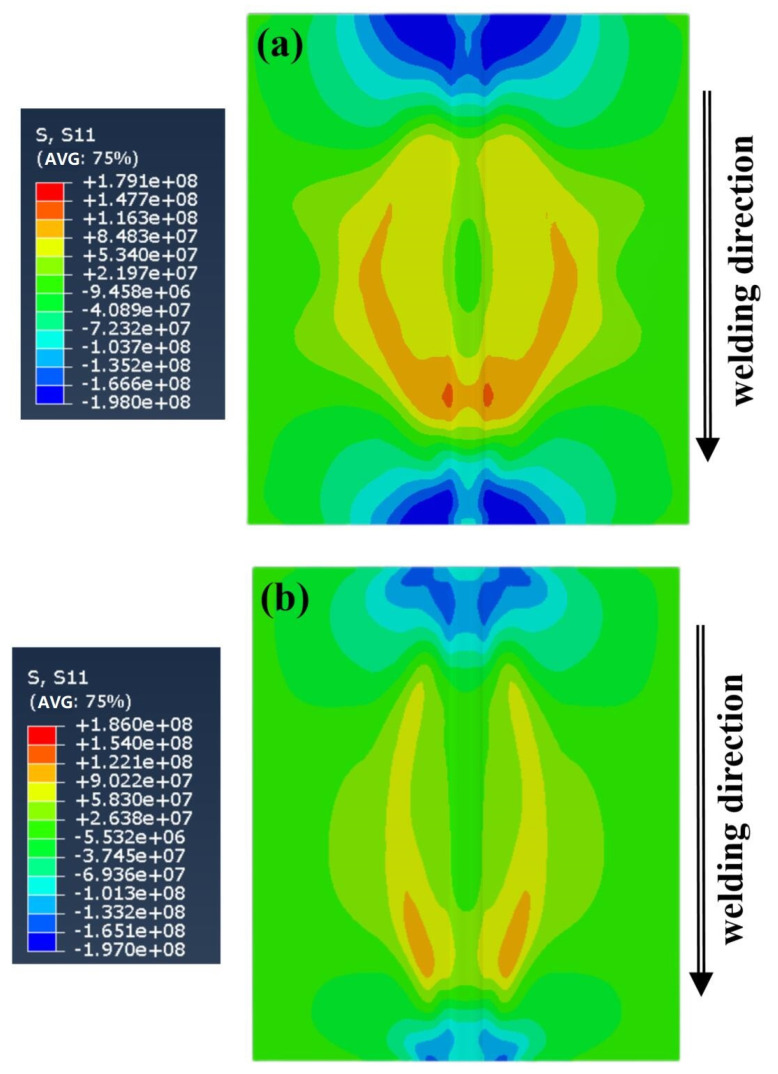
Transverse stress field distribution: (**a**) is low-energy heat source, (**b**) is high-energy heat source.

Figure 13a is a transverse stress field (σ_y_) distribution cloud map of the five-layer welding experiment, and Figure 13b shows a single-layer welding experiment. The transverse stress field distribution trends of the two welding processes are the same. The immediate area of the heat-affected zone of the workpiece shows a tendency toward high tensile stresses, which decrease as they spread to the sides, with the highest stress values located at the bottom of the joint; the arc starting position and the arc extinguishing position show a trend towards compressive stresses. This distribution is because when the heat source leaves and cools to room temperature, the synthesis of transverse and longitudinal shrinkage in the plastic deformation region near the weld causes inward shrinkage at both ends, generating compressive stresses. The rest area generates tensile stresses is what brings the internal ones of the workpiece into balance with residual stresses.

The tensile and compression ranges for the single-layer welding experiment were narrower than for the five-layer welding experiment, indicating a higher predicted processing quality for the single-layer welding experiment. However, the tensile peak at the bottom of the single-layer weld was higher than that of the five-layer weld. This phenomenon is because the heat from each layer of the five-layer weld is superimposed on the previous layer that has already been welded, the workpiece melts to a greater extent, and residual stresses continue to be superimposed and extend throughout the structure. The experimental process of single-layer welding uses a high-energy heat source that acts directly on the bottom liner, which melts at a higher temperature and produces a more prominent stress peak.

**Figure 14:**
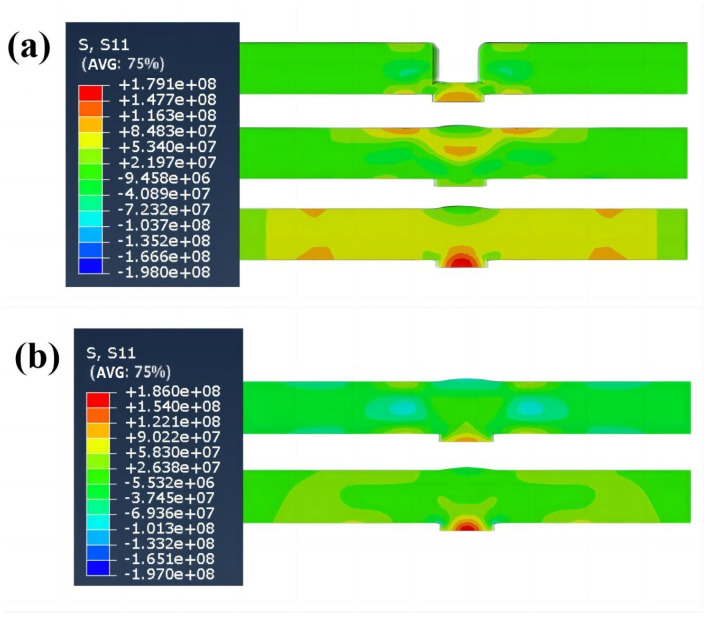
The σ_y_ diagram of the middle along the workpiece B line cutting: (**a**) is the five-layer experiment, (**b**) is the single-layer experiment.

In Figure 14a, at the end of the first layer of the experiment, the bottom part of the workpiece connection is the tensile trend, the upper and lower surfaces on both sides of the molten pool are the tensile trend, and the middle part on both sides of the molten pool is the compression trend. At the end of the fifth layer experiment, the tensile state tends to expand to both sides, and the higher tensile field appears in the middle of the workpiece connection and the upper surface of the heat-affected zone; after the experiment is completed, it is cooled to the final state at ambient temperature. As a result, the middle part of the whole workpiece is stretched, and the highest amount is located at the bottom of the workpiece connection. This stress field distribution trend is due to the repeated heating of the five-layer experiment, the different surface and internal cooling conditions of the workpiece, and the possible superposition of the phase transition process.

In Figure 14b, at the end of the first layer of the experiment, the bottom of the welding track and the upper and lower surfaces on both sides shows a tensile stress trend, and the central area on both sides around it offers a compression trend. After the experiment, it is cooled to the final state at ambient temperature. The highest tensile stress field is at the bottom of the welded track, which is more significant than the highest stress field of the five-layer experiment. Because the high-energy heat source has been acting on the bottom of the track, the thermal expansion and constraint at the bottom are more prominent. However, the overall tensile trend, except at the bottom of the track, is less than five layers of the experiment.

**Figure 15:**
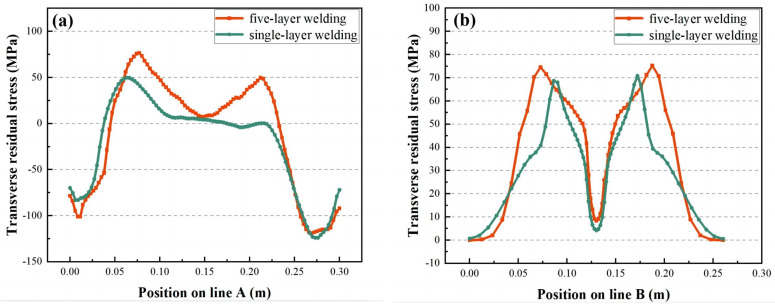
The stress field contrast diagram of high-energy heat source and low-energy heat source: (**a**) is the transverse stress field of A line, (**b**) is the transverse stress field of B line.

Figure 15 shows the trend of σ_y_ distribution in lines A and B under different energy heat source loading. The changing direction of the stress field of the high-energy heat source and the low-energy heat source is consistent and symmetrical about the center of the welded joint, and the overall trend is similar to that of the M type. However, the maximum tensile stress value is significantly lower for the high-energy heat source. The area near the arc starting and extinguishing of the stress field of line A is a compression trend, and the middle part is a tensile trend. The maximum tensile state of approximately 75 MPa and 68 MPa is located in the middle area of 75 mm from the two ends. The stress field in line B is tensile from arc initiation to arc extinguishment. The maximum tensile stress for a single-layer weld is approximately 68 MPa, located in the center region 75 mm from the edge of the workpiece. The maximum tensile stress for a five-layer weld is approximately 75 MPa located in the central region 90 mm from the edge of the workpiece, which is 17% wider than the single-layer weld.

### 4.4. The Influence of the Initial Temperature of the Workpiece on the Residual Stress

To reduce the peak longitudinal residual stress of high-energy heat sources, the longitudinal residual stress field distribution trends of high-energy and low-energy heat sources were made more similar by adopting the method of heating the workpiece before the experiment. This method can reduce the shrinkage stress at the joint of the workpiece and the adjacent matrix material, especially at the high-constraint welding track. Reducing the cooling rate in the critical temperature range thereby avoids excessive hardening and ductility reduction at the joint of the workpiece and the heat-affected zone, increases the cooling time, allows more time for hydrogen to escape from the workpiece, and avoids hydrogen-induced cracks.

**Figure 16:**
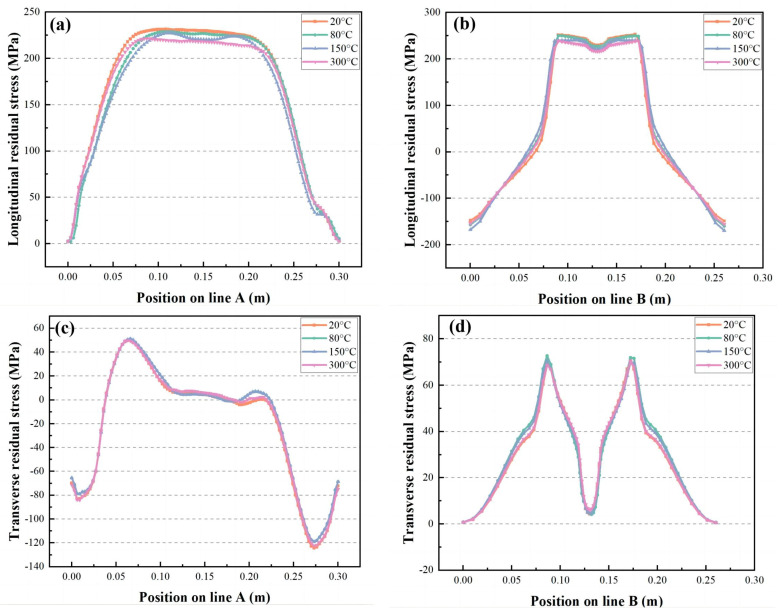
The influence of the initial temperature on the residual stress of A line and B line: (**a**,**b**) represents the longitudinal stress field, and (**c**,**d**) denotes the transverse stress field.

In the two groups of experiments, the longitudinal residual stress of the single-layer experiment is higher than that of the five-layer experiment, and the transverse stress field is the opposite. The five-layer experiment is higher than the single-layer experiment, so it mainly studies the longitudinal stress field this time. In Figure 16a,b, the single-layer experiment taking four different preheating temperatures, the maximum stress distribution trends are 230 MPa and 255 MPa in the longitudinal stress fields of line A and line B at 20 °C. As the temperature increases, the maximum stress decreases. At 300 °C, the top stress distributions of lines A and B are 215 MPa and 220 MPa, 6.5% reduction in line A and 13.7% reduction in line B, almost the same as the five-layer experimental stress field. In Figure 16c,d, different preheating temperatures have virtually no effect on the peak value of the transverse stress field. Therefore, in the high-energy heat source prediction experiment, the highest state of the longitudinal stress field can be reduced by increasing the temperature of the workpiece before welding, and the predicted experimental results of higher weld quality can be obtained.

## 5. Conclusions

In this experiment, the thermal elastic-plastic method is used to predict the changing trend of the residual stress field of a five-layer low-energy heat source and a single-layer high-energy heat source at different experimental temperatures in the ABAQUS simulation application. In addition, the blind hole method detects low-energy heat sources’ longitudinal stress field distribution trend, comparing the predicted results. As a result, the following conclusions can be drawn.

1. The distribution of stress and temperature fields obtained by the blind hole detection technique and the thermocouple measurement method is consistent with the results of the prediction experiments.
2. The trend of longitudinal residual stress and transverse residual stress distribution under the two welding processes is almost identical, but the distribution range of σ_x_ and σ_y_ is smaller in the single-layer high-energy welding experiments.
3. The peak longitudinal residual stress on the upper surface of the workpiece using a high-energy single-layer welding experiment is slightly higher, but the peak transverse residual stress is smaller than the low-energy five-layer welding experiment.
4. Increasing the initial temperature of the workpiece before the experiment can effectively reduce the peak longitudinal residual stress of the high-energy single-layer welding experiment, optimizing the welding quality of this calculation method.
5. The calculation time of the high-energy single-layer welding experiment is 1/4 of that of the low-energy five-layer welding experiment, implying that the use of the high-energy single-layer calculation method can not only improve the weld quality but also reduce the time cost and greatly improve the calculation efficiency of future prediction experiments in the industry under the premise of increasing the preheating temperature of the workpiece.

## Figures and Tables

**Table 1:** Mechanical properties of Q235A and H08Mn2SiA welding wire.

Material Type	Yield Strength/MPA	Tensile Strength/MPA	Modulus of Elasticity/MPA	Poisson Ratio
Q235A	225	450	200	0.3
H08Mn2SiA	420	540	206	0.3

**Table 2:** The composition of Q235A and metal filler.

Material Type	C	Mn	Si	S	P	Cr	Ni	Cu
Q235A	≤0.22	≤1.4	≤0.35	≤0.05	≤0.045	≤0.03	≤0.03	≤0.03
H08Mn2SiA	0.11	1.8–2.1	0.65–0.95	0.03	0.03	0.02	0.005	0.11

**Table 3:** Physical behavior of Q235A.

Temperature/°C	Modulus of Elasticity/GPA	Yield Strength/MPA	Poisson Ratio
20	211	228	0.288
100	209	200	0.291
500	184	150	0.283
700	175	80	0.289
1000	10	30	0.289
1500	10	10	0.289
5000	10	1	0.289

**Table 4:** Thermodynamic properties of Q235A.

Temperature/°C	Thermal Conductivity/W·m^−3^/C	Specific Heat/J·kg^−1^C^−1^	Coefficient of Linear Expansion
20	52	420	11.7
100	51	487	12
500	37	547	13.9
700	32.5	619	14.2
1000	26.2	694	14.8
1500	120	694	14.8
5000	120	694	14.8

## Data Availability

Not applicable.
